# Effect of Acellular Adipose Matrix on Wound Regeneration in a Murine Model

**DOI:** 10.1007/s00266-026-05877-1

**Published:** 2026-05-21

**Authors:** Jaewoo Kim, Vinh Vuong The Tran, Dongjian Li, Jinil Choi, Hak Chang, Ki Yong Hong

**Affiliations:** 1 Department of Plastic and Reconstructive Surgery, Seoul National University College of Medicine, Seoul, Republic of Korea; 2 Department of Plastic and Reconstructive Surgery, SMG-SNU Boramae Medical Center, Seoul, Republic of Korea; 3 Department of Surgery, Hai Phong University of Medicine and Pharmacy, Hai Phong, Vietnam; 4 Department of Plastic and Reconstructive Surgery, Armed Forces Capital Hospital, Seongnam, Republic of Korea; 5 Department of Plastic and Reconstructive Surgery, Seoul National University Hospital, Seoul, Republic of Korea

**Keywords:** Acellular adipose matrix, Decellularized adipose tissue, Wound healing, Wound regeneration

## Abstract

**Background:**

Chronic and acute wounds pose major challenges in clinical practice. Current treatments offer therapeutic benefits but are limited by high cost, technical complexity, and limited availability. The acellular adipose matrix (AAM), derived from decellularized human adipose tissue, contains extracellular matrix components and bioactive molecules that may promote tissue repair and angiogenesis. In this study, we evaluated the therapeutic potential of AAM in promoting wound healing in a murine model.

**Methods:**

Human abdominal adipose tissue was processed into AAM through mechanical, chemical, and enzymatic decellularization. Bilateral full-thickness dorsal wounds (1 cm in diameter) were created in nude mice. The control group received an antibiotic ointment, while the experimental group received AAM. Wound closure was monitored for 16 days. Histological and immunofluorescence analyses were performed to assess epithelialization, collagen remodeling, and angiogenesis. Statistical analysis was performed using the Mann–Whitney U test, with *p* < 0.05 considered significant.

**Results:**

The AAM-treated group exhibited faster epithelial coverage and granulation tissue formation than the control group. The mean healing time was significantly shorter in the AAM group (9.0 ± 1.67 days) compared with controls (12.0 ± 1.79 days). CD31 staining confirmed the presence of dense microvascular networks and significantly enhanced angiogenesis. Collagen fibers were evenly organized without abnormal fibrosis in both groups.

**Conclusions:**

AAM effectively enhanced wound regeneration and angiogenesis in vivo. Its biological activity, accessibility, and potential for cost-effective production suggest that AAM may serve as a clinically applicable “off-the-shelf” biomaterial for wound management in both reconstructive and aesthetic surgeries.

**No Level Assigned:**

This journal requires that authors assign a level of evidence to each submission to which Evidence-Based Medicine rankings are applicable. This excludes Review Articles, Book Reviews, and manuscripts that concern Basic Science, Animal Studies, Cadaver Studies, and Experimental Studies. For a full description of these Evidence-Based Medicine ratings, please refer to the Table of Contents or the online Instructions to Authors www.springer.com/00266.

## Introduction

Wounds impose a substantial burden on national healthcare systems and negatively affect patients’ quality of life. It has been reported that approximately 1.5–2 million Europeans suffer from acute or chronic wounds, while 6.5 million Americans are affected by chronic wounds. The annual cost of wound management is estimated at £5 billion in the United Kingdom, and over $25 billion in the USA. Although the per-patient cost is difficult to estimate, treatment expenses increase substantially in cases involving complications or prolonged hospitalization. In addition to economic costs, patients experience pain, distress, social isolation, anxiety, chronic morbidity, and even mortality [1].

Considerable efforts have been made to accelerate wound healing using hyperbaric oxygen therapy, negative-pressure wound therapy, growth factors, stem cells, and skin substitutes [2]. Skin substitutes are heterogeneous biomaterials that replace the extracellular matrix (ECM) and accelerate wound healing. They can be classified based on their origin—biological or synthetic [3]. Biologically derived substitutes, such as the human acellular dermal matrix, have been used for over two decades to treat acute and chronic wounds, including burns and diabetic foot ulcers [4]. However, human acellular matrices are derived from cadaveric tissues, which can be limited in supply in some countries and may raise concerns regarding immunogenicity and pathogen transmission [5]. Moreover, their manufacturing process is labor- and time-intensive, particularly during hair removal and sterilization, leading to high production costs and restricted accessibility for patients.

Adipose tissue is one of the most abundant, expandable, and safely harvested tissues in the human body. Recently, the acellular adipose matrix (AAM)—an ECM isolated from adipose tissue—has been introduced as a promising alternative. Adipose tissue undergoes mechanical, chemical, and biological processes to completely remove cellular components [6]. AAM retains ECM components such as collagen, elastin, proteoglycans, and laminin and acts as a biological scaffold that enhances neovascularization and re-epithelization [7], thus promoting wound healing. Moreover, AAM can be easily applied to wounds of various sizes and depths. In this study, we aimed to investigate the therapeutic effects of AAM on wound healing using a murine model.

## Methods

### Adipose Tissue Harvesting and Acellular Adipose Matrix Processing

The study protocol was approved by the Institutional Review Board of our institution [IRB No: H-2304-033-1420). Abdominal adipose tissue samples were obtained from discarded tissues of patients aged 30‒40 years who underwent transverse rectus abdominis myocutaneous flap surgery for breast reconstruction.

Fat tissue was converted into AAM through mechanical, chemical, and biological processing following a previously described protocol. Briefly, adipose tissue underwent five freeze–thaw cycles and was centrifuged at 1,500 rpm for 10 min. After washing, the fatty portion was discarded, and the tissue was treated with 99.9% isopropanol for 8 h for polar solvent extraction. The processed tissue was then incubated in a solution of 0.05% trypsin, 0.05% EDTA, 10 ng/ml DNase I (Sigma-Aldrich; Merck KGaA, Darmstadt, Germany), and 10 ng/ml RNase (Sigma-Aldrich; Merck KGaA) for 2 h with gentle rotation at 37˚C. Finally, the tissue was washed and treated with 1% penicillin-streptomycin (Sigma-Aldrich; Merck KGaA) for 12 h at 4˚C and then dried under UV light for 4 h at room temperature.

### Animal Models and Procedures

Ten-week-old athymic nude mice (weight: 20–25 g) were purchased from Koatech (Gyeonggi-do, South Korea). A total of six mice were included in the study. The mice were housed in a temperature-controlled environment at 24±2 °C with an artificial 12-hour light/dark cycle. All applicable institutional and/or national guidelines for the care and use of animals were followed.

**Fig. 1:**
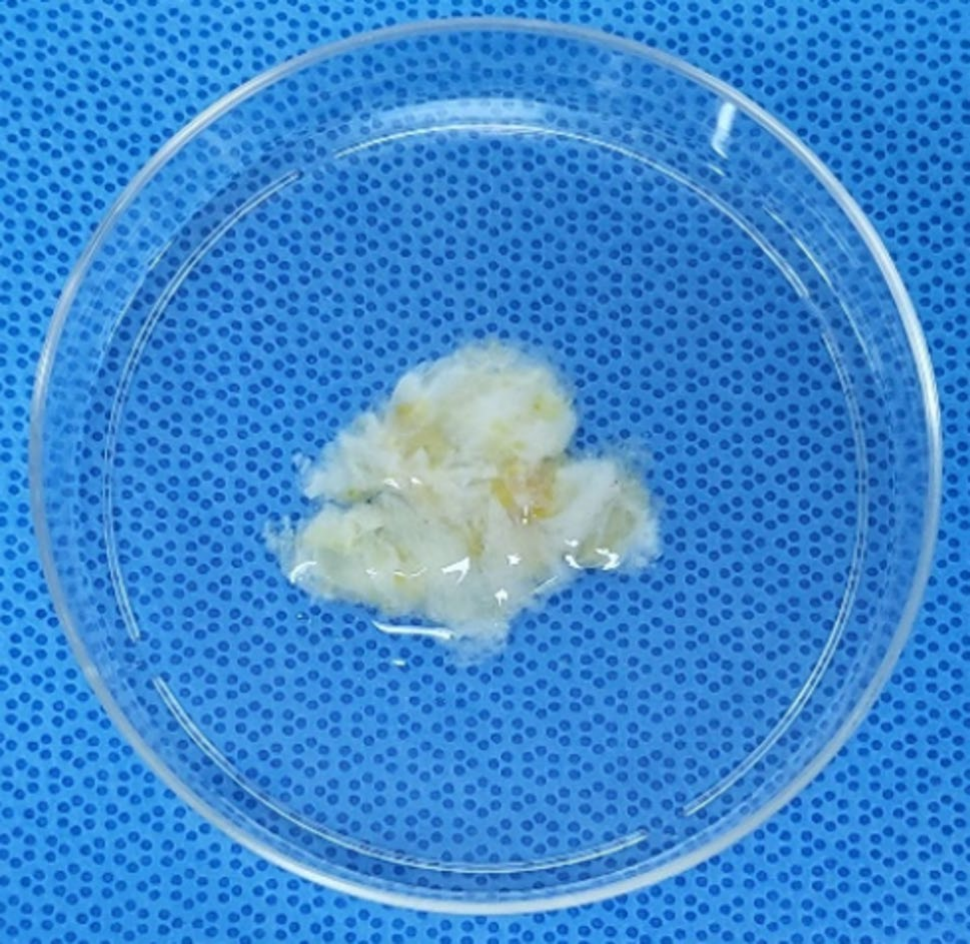
Manufactured AAM in a sterile Petri dish

Two circular full-thickness skin wounds (1 cm in diameter) were created on the dorsal surface of each mouse. In each animal, one wound was randomly assigned to receive AAM treatment, while the contralateral wound served as the control and received conventional antibiotic ointment, allowing within-animal comparison. To minimize wound contraction, a custom-made silicone splint ring (1 cm inner diameter, 1 mm thickness) was placed around each wound and secured to the surrounding skin with four interrupted 5-0 nylon sutures. The control wound received conventional antibiotic ointment, whereas the contralateral wound was treated with AAM (Fig. 1). All wounds were then covered with a sterile polyurethane foam dressing and fixed using Peha-haft® cohesive elastic tape (Hartmann, Germany) to maintain stability.

Animals were monitored every alternate day to ensure proper wound coverage and general health. Wound-healing progression was documented by digital photography on postoperative days 0, 2, 4, 6, 8, 10, 12, 14, and 16. At each time point, wound size, depth, and epithelization status were evaluated on each side. On day 16, the wound area, including the scar and marginal skin, was harvested for histological and immunofluorescence analyses.

### Histomorphometric Analysis

Tissue samples were fixed overnight in 4% paraformaldehyde (BD Biosciences), dehydrated, and embedded in paraffin. Sections (4-μm thick) were prepared for hematoxylin–eosin and Masson’s trichrome staining. Images were captured using a light microscope (Nikon ECLIPSE Ts2; Nikon, Tokyo, Japan).

Unstained slides were deparaffinized with xylene, rehydrated, and boiled in citrate buffer (Agilent Technologies, Santa Clara, Calif.) for antigen retrieval. Samples were then blocked with 5 % goat serum (Jackson ImmunoResearch, West Grove, Pa.) for 1 h and incubated overnight at 4 °C with primary antibodies. After washing, the samples were incubated with secondary antibodies for 2 h at room temperature. The slides were then counterstained with 4’,6-diamidino-2-phenylindole to visualize nuclei. The primary antibody used was rabbit anti-CD31 (PA5-16301; Invitrogen), and a fluorescein-conjugated goat anti-rabbit secondary antibody was applied. Immunofluorescent images were obtained using a confocal microscope (SP8; Leica, Wetzlar, Germany). Five random fields from each sample were analyzed using ImageJ (Fiji; National Institutes of Health, Bethesda, MD.).

### Statistical Analysis

Data were analyzed using the nonparametric Mann–Whitney U test, as the sample size was small, and the data were not normally distributed. All statistical analyses were performed using R Version 4.0.3, and *p* < 0.05 was considered statistically significant.

## Results

### AAM Fabrication

**Fig. 2:**
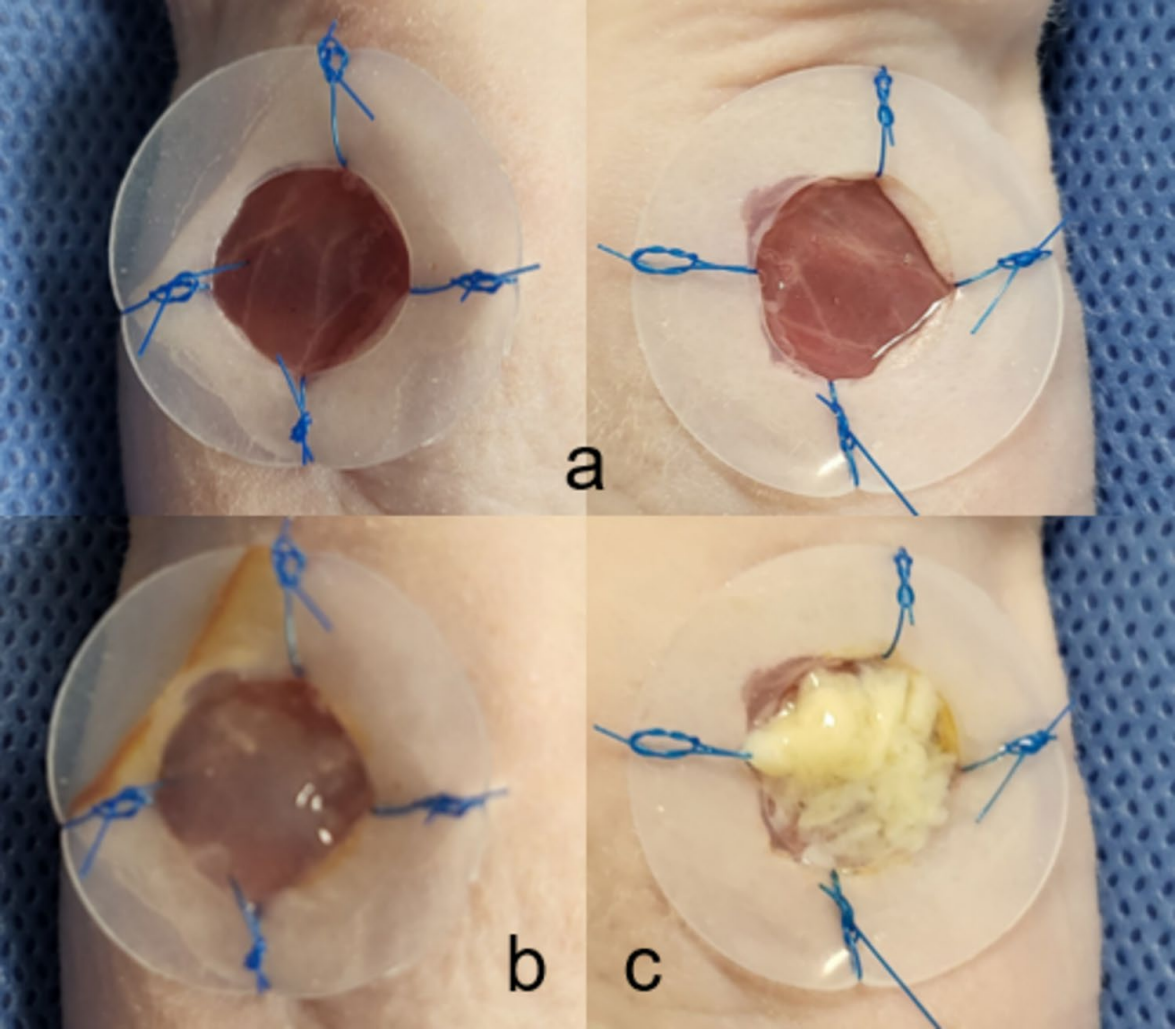
Preparation of the skin defect wound-healing model using a silicone ring splint **a** and conventional ointment **b** or AAM **c** was applied on day 0

Human AAM was manufactured using a modified protocol developed in our laboratory [8]. The freeze–thaw cycle was repeated only five times because previous studies showed that more than five cycles did not enhance the effect but could damage the AAM microstructure. The resulting AAM appeared as a white fibrous tissue, reflecting the collagen-rich composition of the adipose tissue extracellular matrix (ECM) (Fig. 2).

### Macroscopic Observation of Wound Regeneration

Wound evaluation was performed every 2 days until postoperative day 16. During the early phase (days 0–7), no remarkable macroscopic differences in wound size or depth were observed between the AAM-treated and control groups. The applied AAM remained attached to the wound bed throughout this period and was kept in situ during evaluation to avoid interference with the healing process.

**Fig. 3:**
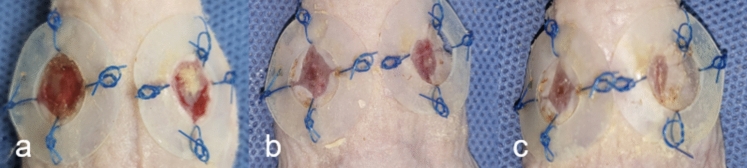
Macroscopic observation of the skin defect wound-healing model. The conventional dressing group is shown on the left and the AAM-treated group on the right at postoperative days 8 **a**, 12 **b**, and 16 **c**.

From postoperative day 8 onward, distinct differences in wound closure became apparent between the two groups. Wounds treated with AAM showed faster granulation tissue formation and earlier epithelial coverage than those in the control group (Fig. 3). The primary macroscopic parameter for comparison was time at which the full-thickness wound was completely covered by new epithelial tissue.

**Fig. 4:**
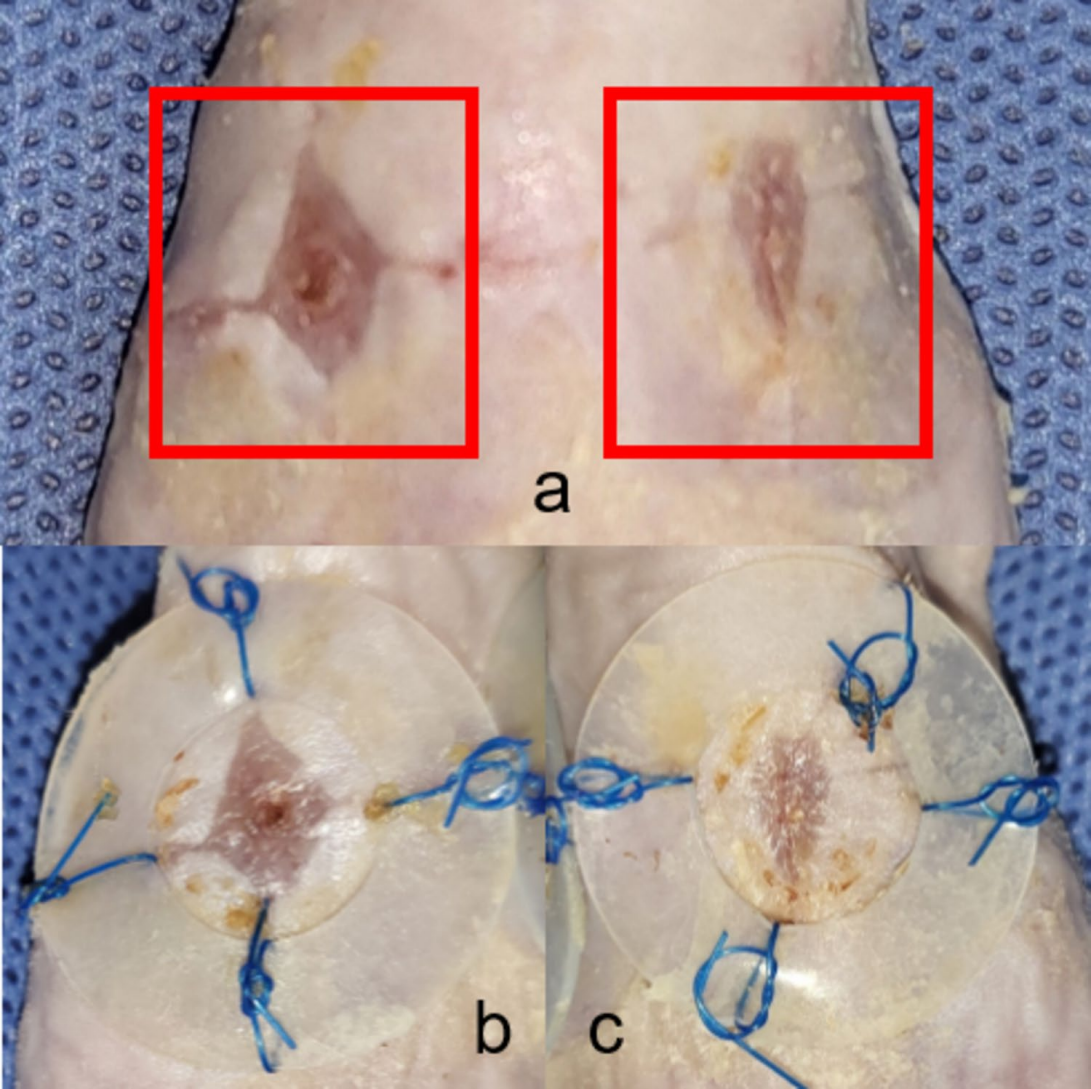
En bloc excision of the wound bed on day 16 **a** in the conventional dressing group **b** and AAM dressing group **c**

Quantitative analysis showed that the mean healing time was significantly shorter in the AAM group (9.00 ± 1.67 days) than in the control group (12.00 ± 1.79 days) (Mann–Whitney U test, *p* = 0.026; n = 6 wounds per group) (Table 1) (Fig. 4). These findings indicated that AAM effectively accelerates wound regeneration in a murine model.

**Table 1:** Summary of wound-healing time and CD31-positive area in control and AAM-treated wounds

Parameter	Control (mean ± SD)	AAM (mean ± SD)	p value
Healing time (days)	12.0 ± 1.79	9.0 ± 1.67	0.026
CD31-positive area (%)	0.500 ± 0.162	0.811 ± 0.167	0.047

Data are presented as mean ± standard deviation. CD31-positive area (%) was quantified using ImageJ analysis of immunofluorescent images. Statistical comparisons were performed using the Mann–Whitney U test. n = 6 wounds per group for wound-healing time and n = 5 wounds per group for CD31 analysis.

### Histologic Findings of Wound regeneration

**Fig. 5:**
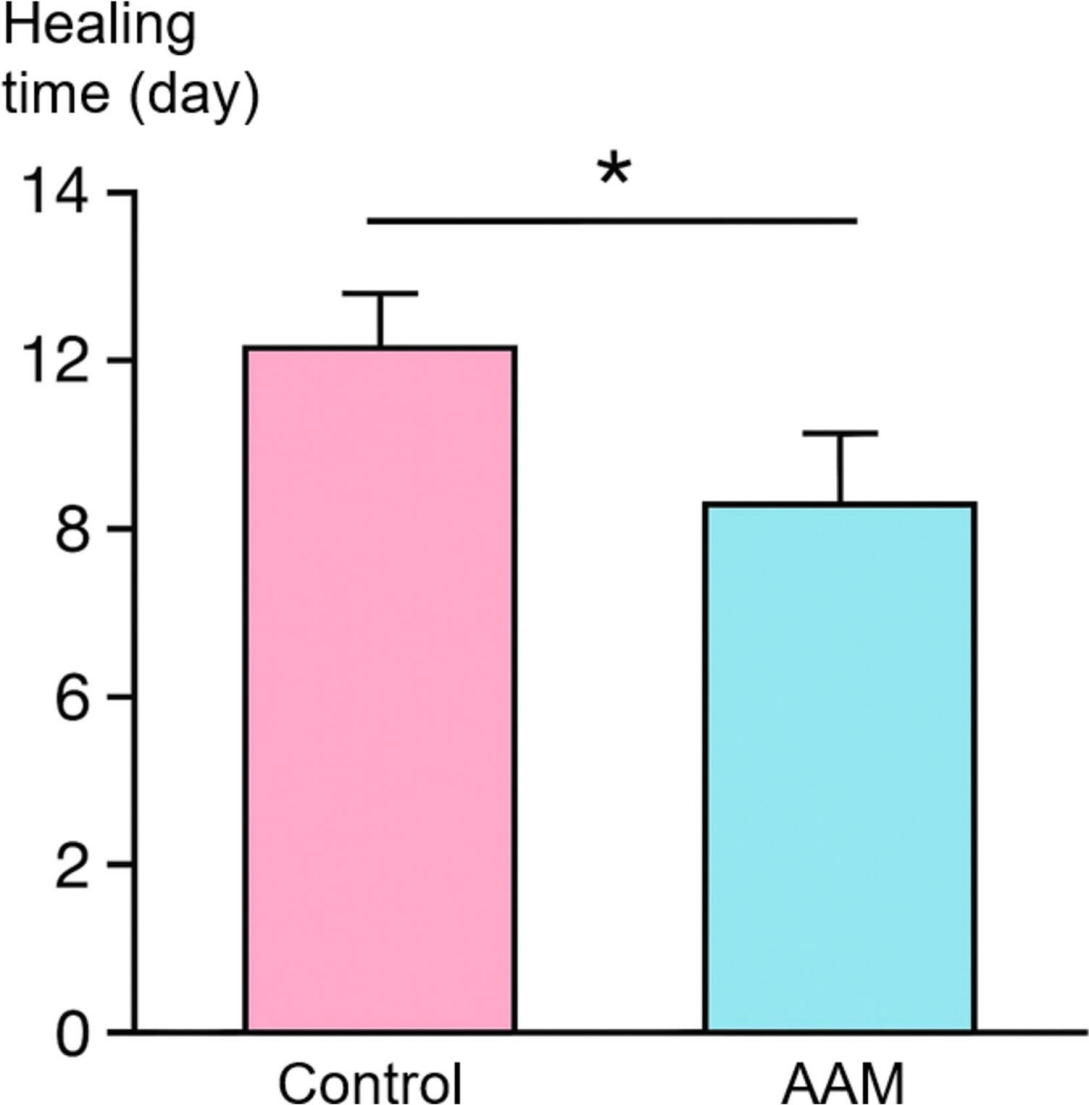
Comparison of mean wound-healing times between the conventional dressing and AAM dressing groups showed a statistically significant difference. The Mann–Whitney U test was used for analysis; data are presented as mean ± standard deviation; n = 6 wounds per group

On postoperative day 16, wound tissues were harvested as a single piece for histological analysis (Fig. 5). Hematoxylin and eosin (H&E) and Masson’s trichrome staining were used to evaluate epithelialization, inflammation, collagen remodeling, and neovascularization.

**Fig. 6:**
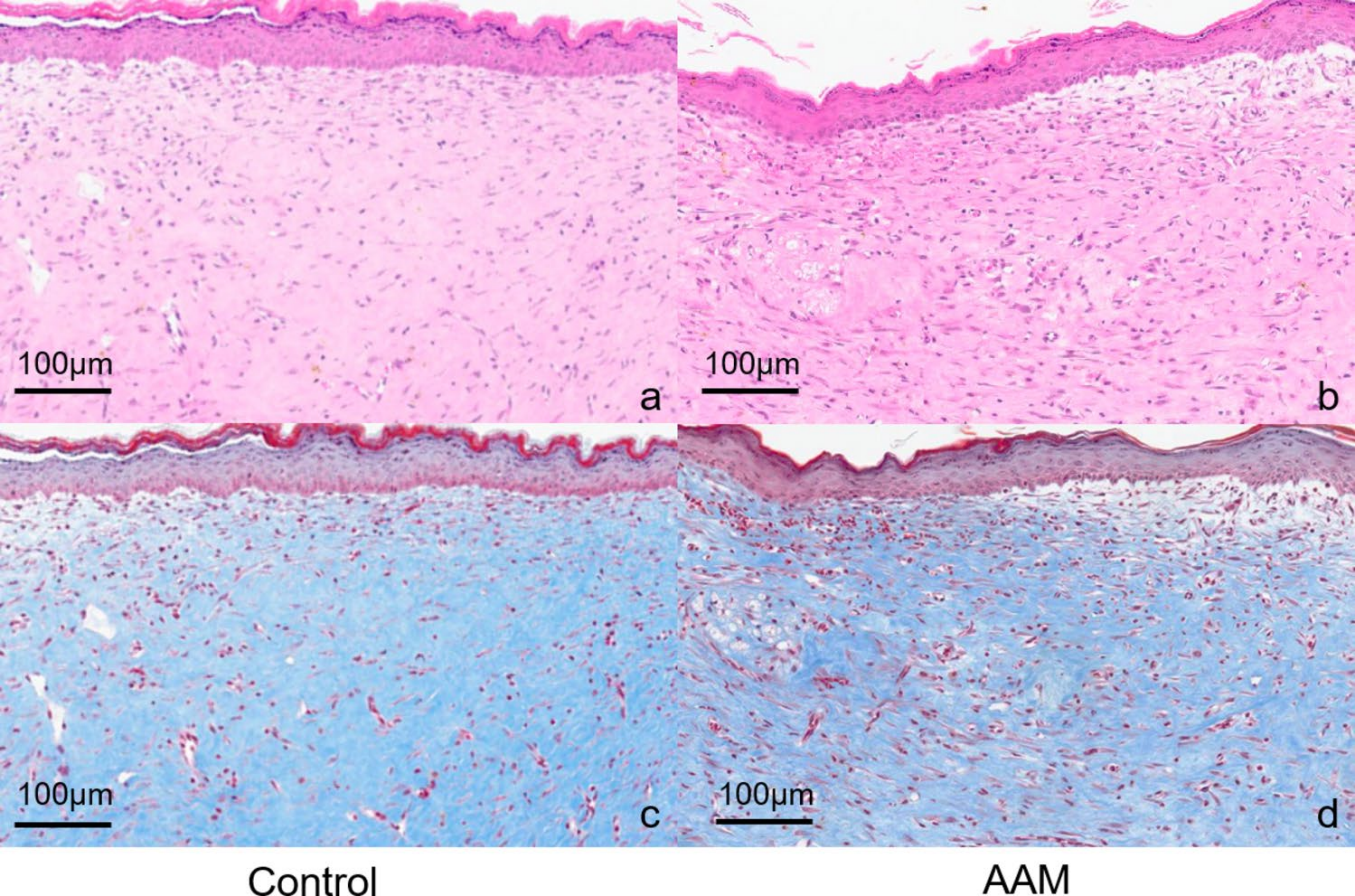
Histologic findings of en bloc excised wound beds on day 16 from the conventional** a, b** and AAM dressing groups **c, d**. Scale bar, 100 µm

Both the AAM and control groups exhibited complete epithelial coverage across the wound bed, confirming full re-epithelialization. Inflammatory cell infiltration was minimal and comparable between the two groups. Collagen fibers were evenly distributed within the granulation tissue, with no apparent difference in collagen density or organization. The AAM group, however, showed a higher number of new blood vessels (Fig. 6).

### Analysis of Angiogenesis

**Fig. 7:**
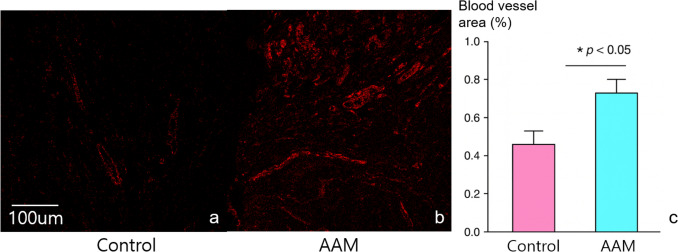
Immunofluorescent staining of endothelial cells in wound beds on day 14 from the conventional dressing **a** and AAM dressing groups **b**. Red signals represent CD31-positive cells, indicating angiogenesis. The percentage of CD31-positive area in the AAM dressing group differed significantly from that in the conventional dressing group **c**. The Mann–Whitney U test was used for analysis; data are presented as mean ± standard deviation; n = 5 wounds per group; scale bar, 100 µm

To assess neovascularization within the regenerated wound tissue, CD31 immunofluorescence staining was performed on five randomly selected sections from each group on postoperative day 16. CD31-positive microvessels were primarily distributed along the granulation tissue beneath the neoepithelium (Fig. 7).

Qualitatively, the AAM-treated group demonstrated a denser and more organized microvascular network than the control group. Quantitative analysis of the CD31-positive area confirmed that angiogenesis was significantly enhanced in the AAM group. The percentage of CD31-positive area was 0.811 ± 0.167% in the AAM-treated group and 0.500 ± 0.162% in the control group. Statistical comparison using the Mann–Whitney U test revealed a significant difference between the two groups (*p* = 0.047; n = 5 wounds per group) (Table 1) (Fig. 7).

These findings indicated that AAM application effectively promotes angiogenesis during wound healing in a murine model.

## Discussion

AAM, derived from decellularized adipose tissue, is a promising ECM-based material for tissue repair and regeneration. Previous studies have shown that decellularized adipose tissue preserves essential ECM components such as collagen, elastin, and laminin, which provide a favorable environment for cell migration, proliferation, and angiogenesis [5, 6]. AAM also retains bioactive factors, such as VEGF, that promote neovascularization and tissue repair [4, 9]. Recent reviews have reported that decellularized ECM accelerates wound closure and enhances skin regeneration by supporting epithelial growth and vascular formation [2, 10, 11]. However, most of these studies were preliminary and involved small sample sizes; thus, preclinical data under clinically relevant conditions remain limited [1, 3, 7].

In this study, we evaluated the healing effects of human-derived AAM in a mouse full-thickness wound model, aiming to bridge basic research and potential clinical applications. The AAM-treated group healed faster and exhibited greater blood vessel formation than the control group. These findings confirm the regenerative capacity of AAM and support its potential as a ready-to-use biomaterial for wound treatment [6, 9, 11].

The wound-healing effects of AAM likely arise from both its structural and biological properties. Structurally, AAM preserves the native ECM framework, providing mechanical support and facilitating fibroblast migration and granulation tissue formation [5]. Biologically, growth factors and glycosaminoglycans within the ECM may be gradually released, promoting angiogenesis through *VEGF/VEGFR* and *PI3K–Akt* signaling pathways [12, 13]. This hypothesis is consistent with our finding of increased CD31-positive microvessels in the AAM group. Moreover, because AAM is injectable and flexible, it conforms well to irregular wound surfaces, maintaining a moist and active environment that promotes epithelialization and tissue regeneration [6].

Clinically, AAM offers several advantages over traditional materials such as acellular dermal matrix (ADM). Although ADM has proven safety and efficacy, it is expensive, time-consuming to prepare, and reliant on limited donor availability. In contrast, adipose tissue is easy to obtain, abundant, and ethically safe. This makes AAM production easier and cheaper. AAM can also be manufactured in various forms to fit wounds of different shapes and depths. An ideal wound dressing should be both effective and economical [14], and AAM meets these criteria, representing a practical option for managing complex or chronic wounds.

Despite the promising results, this study had some limitations. First, the number of animals was small, and the observation period was limited to 16 days, which may not reflect long-term healing and tissue remodeling. Second, the molecular mechanisms underlying AAM-induced angiogenesis were not fully explored. Future research should assess the expression of angiogenic factors such as *VEGF* and *TGF-β* at both the gene and protein levels. Third, although decellularization minimizes immunogenicity, mild immune responses from residual DNA or proteins may still occur [15].

Future studies should compare AAM with clinically used synthetic collagen dressings in normal, diabetic, and ischemic wound models to determine relative advantages and further validate its wound-healing efficacy [16]. Additionally, combining AAM with negative-pressure wound therapy [17] may enhance its regenerative potential.

From a translational perspective, the biological activity and accessibility of AAM support its potential as a clinically applicable biomaterial. These findings suggest that AAM may serve as a promising “off-the-shelf” option for wound management in reconstructive and aesthetic clinical practice.

## Conclusion

AAM effectively promoted wound healing by accelerating tissue regeneration and angiogenesis in mice.
